# Posterior scleral reinforcement for the treatment of myopic traction maculopathy

**DOI:** 10.1186/s12886-022-02497-6

**Published:** 2022-06-21

**Authors:** Qing He, Xiu Wang, Qianhui Shi, Caiyuan Xie, Anquan Xue, Ruihua Wei

**Affiliations:** 1 Tianjin Key Laboratory of Retinal Functions and Diseases, Tianjin Branch of National Clinical Research Center for Ocular Disease, Eye Institute and School of Optometry, Tianjin Medical University Eye Hospital, No.251, Fukang Road, Nankai District, Tianjin, 300384 China; 2 The Eye Hospital, School of Ophthalmology and Optometry, Wenzhou Medical University, Wenzhou, Zhejiang China

**Keywords:** Posterior scleral reinforcement, Myopic traction maculopathy, Pathologic myopia

## Abstract

**Background:**

This study aimed to investigate the clinical effectiveness of posterior scleral reinforcement(PSR) for the treatment of myopic traction maculopathy (MTM).

**Methods:**

This was a prospective study of 32 eyes from 20 patients with MTM treated with PSR using genipin-cross-linked donor sclera. The length of the scleral strip used for the surgery was designed to be 1.5-times the axial length of the eye, whereas its width was 0.4-times the axial length of the eye. The optical coherence tomography images, spherical equivalent of refractive error, axial length, best corrected visual acuity, electroretinogram findings, and intraocular pressure of the patients were assessed postoperatively.

**Results:**

The mean duration of follow-up was 17.80 ± 8.74 months. The differences between the spherical equivalent of refractive error, best corrected visual acuity, axial length, and electroretinogram findings recorded preoperatively and those measured postoperatively were statistically significant (*p* < 0.05). The final reduction in axial length was 1.64 ± 0.85 mm. At the end of the follow-up, optical coherence tomography showed essential foveal reattachment in 30 eyes (93.75%), partial reattachment in two eyes (6.25%), and closure of macular holes in seven eyes (77.78%). No retinal detachment, vitreous haemorrhage, or other serious complications occurred following the surgery.

**Conclusions:**

Posterior scleral reinforcement with genipin-cross-linked sclera showed safe and effective outcomes for the treatment of MTM during a follow-up period of at least one year.

**Trial registration:**

11\12\2018, ChiCTR1800020012.

**Supplementary Information:**

The online version contains supplementary material available at 10.1186/s12886-022-02497-6.

## Background

Myopic traction maculopathy (MTM) is one of the most common complications of pathological myopia and was first described by Panozzo and Mercanti [1]. It is a major cause of decreased vision in patients with myopia [1, 2]. Myopic foveoschisis (MTM), retinal detachment (RD), lamellar macular hole (LMH), and full-thickness macular hole (FTMH), with (MHRD) or without RD, are the different stages of MTM [1].

A variety of surgical options are currently available for the treatment of MTM [3]. These include intraocular surgeries—pars plana vitrectomy (PPV) combined with silicone oil or gas tamponade [4] with or without internal limiting membrane peeling—and extraocular surgeries—posterior scleral reinforcement (PSR) [5] and combined surgery [6]. However, there is increasing evidence [7] that the outcomes of a single intraocular surgery are often unsatisfactory. Conart et al. reported that 8.5% of 47 highly myopic eyes with macular detachment treated with PPV had recurrence after surgery [8]. Besides, PPV is a complicated and potentially damaging intraocular surgery that is associated with a risk of complications after combined silicone oil filling, such as glaucoma, cataracts, and risks associated with silicone oil removal.

Although MTM is primarily associated with posterior scleral staphyloma (PS) [9], its pathogenesis remains unknown. Kosei et al. [10]found that eyes with PS were more likely to develop retinal vascular microfolds in the outer macular schisis, perhaps this may be related to the backward vertical force of the PS. Zhang et al. [11]also considered that MTM develops due to the pulling action of posterior scleral staphyloma because the retina lacks the ductility to match the expansion of the sclera. PPV relieves the tangential and centrifugal traction forces caused by the vitreous cortex without tackling the persistently expanding posterior scleral staphyloma [12, 13], which is the fundamental reason for its poor clinical outcome. In contrast, PSR [13, 14]can shorten a patient’s axial length (AL) and reinforce the posterior scleral staphyloma, while reducing the retinal traction caused by the vitreous and posterior scleral staphyloma. Furthermore, several studies [7, 14–18] have indicated that the efficacy of PSR for the treatment of MTM is superior to that of PPV. However, differences in the material and shape of the scleral strip used can affect the outcome of the surgery.

Several studies [19, 20] have focused on the clinical outcomes of posterior scleral reinforcement surgery, most of which evaluate the postoperative retinal structural reattachment. The purpose of this study was to investigate the effectiveness and safety of PSR for the treatment of MTM by evaluating both the structure and function of the retina.

## Methods

This was a prospective study of 32 eyes from 20 patients with MTM who were treated using PSR at the Tianjin Medical University Eye Hospital. Ethical approval for this study was obtained from the Medical Ethics Committee of Tianjin Medical University Eye Hospital. The study procedures adhered to the tenets of the Declaration of Helsinki. All patients included in this study provided written informed consent after thorough discussions on the potential benefits and risks of PSR combined with vitrectomy.

Patients with severe cataracts that fundus cannot be visualized, patients with ocular trauma, and with choroidal neovascularization were excluded from this study. All patients with MTM were assessed postoperatively during follow-up visits at 1, 3, 6 months, and 1 year or longer. The preoperative and postoperative examinations included slit lamp examination, measurement of best corrected visual acuity (BCVA) (Logarithm of the Minimum Angle of Resolution, logMAR), refraction, measurement of intraocular pressure (IOP), measurement of axial length (AL), optical coherence tomography (OCT), and electroretinogram (ERG). AL was measured using Lenstar LS900 (Haag-StreitAG, Switzerland), and the average of three successive measurements was calculated and recorded. OCT (OPTOVUE, Colin, USA) was used for fundus examination; the radial scan was performed with the central macular recess as the centre, and the diameter of the scan was 6 mm. The average of the maximum length of the schisis cavity in 12 directions within the scan range was measured, and the reduction of the schisis cavity was calculated from this average. MTM included different OCT presentations, which needed to be classified in more detailed ways [21]. We used the MTM Staging System (MSS) to evaluate the patient’s preoperative OCT [22], which had good reproducibility and consistency[23]; stage 1 is the inner maculoschisis or inner-outer maculoschisis, stage 2 is a predominantly outer maculoschisis (O-MS); stage 3 is a combination of maculoschisis and foveal detachment (MS-MD); stage 4 is macular detachment without schisis (MD). The evaluation criteria for retinal restoration were as follows [5]: an 80% or more reduction of the retinal schisis cavity was considered essential reattachment (ER), a 40%-79% reduction was considered partial reattachment (PR), and a reduction of less than 39% was considered as not reattached (NR). ERG results were examined using a retinal current meter (ESPION, Diagnosys, USA), and a-wave and b-wave amplitudes were recorded.

### Surgery procedure

All the surgeries were performed by the same experienced doctor under a microscope. All the patients signed a consent form before the surgery. The strips used for the surgery were homogeneous sclerae that were cross-linked and rigorously sterilised using 0.1% genipin [24]. Each strip was shuttle-shaped, 1.5 times as long as the AL of the patient’s eye, and 0.4 times as wide as the AL of the eye. The intraoperative shortening of the AL was expected to range from approximately 2–3 mm. The details of the surgical technique of the PSR have been published previously [25]. After general anesthesia was induced, the bulbar conjunctiva was cut at 210° along the corneal limbus, with the inferior temporal aspect of the eye as the centre. The lateral rectus and inferior rectus traction lines were made, the strips were drawn inwards and upwards and passed through the inferior oblique, lateral rectus, and inferior rectus muscles in turn. 5–0 non-absorbable sutures (Alcon) were fixed to the equatorial anterior sclera between the inferior and medial recti muscles, and the lateral temporal end was fixed to the equatorial sclera between the superior and lateral recti muscles. Before fixation, the anterior chamber was punctured using a 5-gauge needle to release two to four drops of atrial fluid to balance the intraocular pressure. Before the procedure was completed, the strip was checked to ensure that it was in the correct position and orientation. The posterior pole was examined using indirect ophthalmoscopy after pupil dilation to confirm that the optic nerve and major vessels were normal and the macula was mildly elevated. The conjunctival incision was closed using 8–0 absorbable sutures coated with antibiotic eye ointment and bandaged.

### Statistical analysis

Statistical analysis was performed using SPSS software (version 23.0; StataCorp LP, College Station, TX, USA). Data for continuous variables were expressed as mean ± standard deviation. Preoperative and postoperative LogMAR BCVA, ERG, AL, and IOP were compared using paired t-test. *P* < 0.05 indicated a statistically significant difference.

## Results

The patients’ demographic data are presented in Table 1. The participants included three men and 17 women, with a mean age of 51.20 ± 14.82 years. The mean duration of follow-up was 17.80 ± 8.74 months.

**Table 1:** Preoperative data of the patients who underwent PSR for MTM versus their postoperative data recorded at the last follow-up

No	Sex	Age	Eye	Pre-operative						Post-operative							Follow-up (months)
				SE (D)	BCVA	AL (mm)	OCT (MSS)	ERG a(uV)	ERG b(uV)	SE (D)	BCVA	AL (mm)	OCT (retinal restoration)	OCT (MH)	ERG a(uV)	ERG b(uV)	
1	F	31	OD	-7.5	0.1	31.98	2a	32.79	75.28	-6	0.1	31.03	ER	-	42.81	146.7	36
			OS	-13	0.4	33.51	2a	20.61	61.68	-5.5	0.4	31.55	ER	-	48.43	124.9	36
2	F	18	OD	-14.5	0.1	28.26	2a	34.68	60.2	-11.5	0.1	27.05	ER	-	36.8	99.34	12
			OS	-11.5	0.4	27.25	2a	29.13	64.06	-10	0	26.47	ER	-	45.61	111.6	12
3	F	34	OD	-4.5	0.2	31.4	2a	39.85	60.29	-4	0.1	31.31	ER	-	65.2	123.1	13
4	F	40	OD	-16	1.3	30.83	3aO	24.14	66.61	-2	1	30.06	PR	-	36.42	96.65	12
5	F	59	OS	-14	0.5	28.49	2b	34.09	94.53	-12	0.5	26.61	ER	closure	43.57	105.1	42
6	M	33	OS	-7.75	0.2	32.79	2b	24.12	114.9	-6.5	0.3	31.96	ER	closure	38.07	57.23	12
7	F	53	OD	-2.25	0.9	30.68	2c	32.82	49.08	-0.75	0.4	28.88	ER	FTMH	39.24	101.7	12
			OS	-2.5	0.4	29.29	1b	49.7	56.15	-0.25	0	27.73	ER	closure	42.91	99.42	12
8	F	69	OD	-24.5	1.6	30.93	2b	22.57	38.77	-2.5	0.7	27.44	ER	closure	26.92	45.52	14
9	F	77	OD	-4	1.3	32.17	3aO	30.75	47.34	-1	1	29.66	ER	-	49.04	51.42	12
			OS	-3.5	1	30.89	2c	90.96	128.9	-0.25	0.3	28.48	ER	FTMH	110.5	157.1	12
10	F	53	OD	-13.75	1.6	27.17	3a	58.07	107.9	-6.25	1.6	25.4	ER	-	51.41	106.9	12
			OS	-17	0.5	29.18	2a	28.6	109.1	-12	0.3	27.35	ER	-	45.56	124	12
11	F	60	OD	-24	1.6	30.79	4b	21.17	34.55	-22.5	1.6	27.54	ER	closure	29.99	66.55	29
			OS	-22.5	0.7	28.79	2a	25.66	69.78	-17	0.5	26.89	ER	-	35.24	138.2	29
12	F	61	OD	-12	0.3	28.72	2a	33.5	93.04	-3.5	0.2	27.14	ER	-	21.45	41.15	24
13	F	58	OD	-13.75	0.5	26.49	2a	28.44	47.63	-5.25	0.3	25.45	ER	-	43.24	140	12
14	F	59	OD	-21.5	0.5	29.59	2a	44.97	99.31	-12	0.4	26.62	ER	-	57.35	121.35	12
			OS	-22.25	1.3	31.02	2c	33.46	80.78	-14	1.3	28.35	ER	closure	9.12	102.44	12
15	F	64	OD	-24.75	1.6	32.03	2b	27.63	61.51	-20	1.6	30.31	ER	closure	26.34	97.68	12
			OS	-26.5	1	31.79	2a	19.38	40.01	-17	0.8	29.01	ER	-	47.26	89.56	12
16	F	49	OD	-11.75	0.7	30.13	2a	32.24	105	-17	0.8	29.41	ER	-	47.69	98.77	12
			OS	-3.75	0.7	29.85	2a	35.96	104.4	-1.5	0.4	28.13	ER	-	46.37	103.24	12
17	M	49	OD	-26.5	0.8	32.18	3a	39.32	130.8	-3.25	0.2	30.63	ER	-	26.79	85.85	14
			OS	-26.5	0.8	31.33	3a	34.12	117.3	-2.5	0.7	29.48	ER	-	22.65	66.94	14
18	M	31	OD	-21	0.2	31.62	2a	38.98	60.53	-20	0.2	31.57	ER	-	45.67	89.79	25
19	F	62	OD	-21	1.6	30.3	2a	15.51	47.82	-18.5	1.5	28.46	ER	-	21.47	44.6	20
			OS	-14.5	0.4	27.52	2a	13.38	52.64	-11	0.3	26.92	ER	-	34.24	55.82	20
20	F	59	OD	-23	1	27.87	3a	39.42	112.1	-21	0.7	26.91	PR	-	28.22	61.85	19
			OS	-24.5	1.3	27.02	3a	31.42	94.67	-11	1.3	25.61	ER	-	24.77	55.22	19

*SE* Spherical equivalent, *BCVA* Best corrected vision acuity, *AL* Axial length, *MH* Macular hole, *ER* Essentially reattached, PR Partially reattached, *ERG* Electroretinogram, *OD* Right eye, *OS* Left eye, *OCT* Optical coherence tomography

**Fig. 1:**
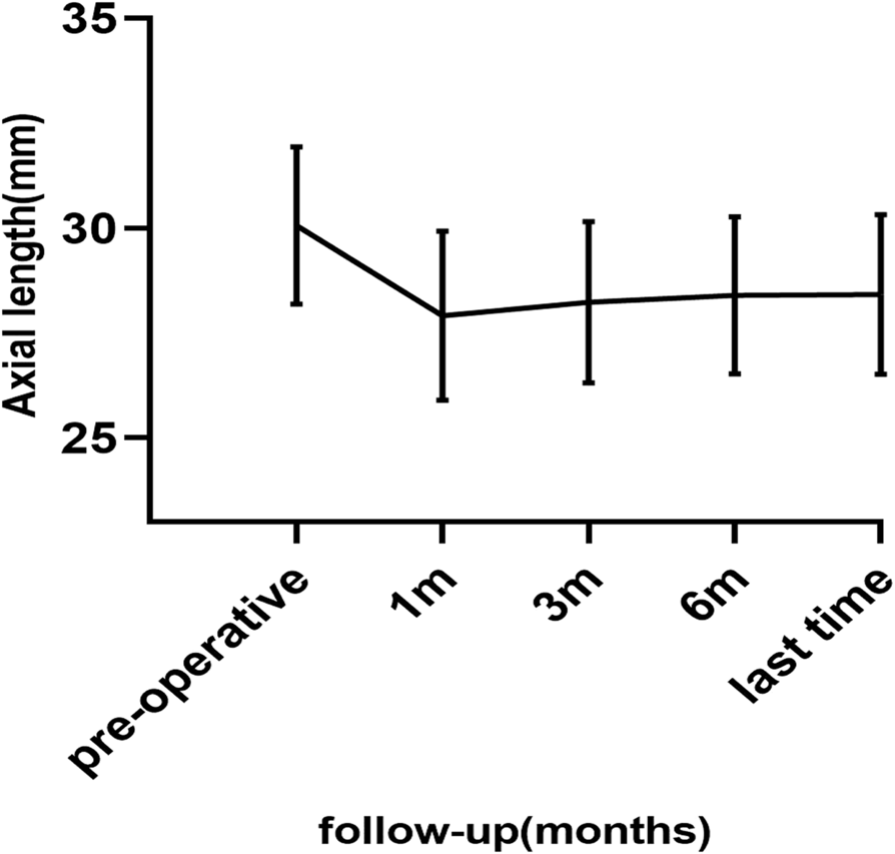
Changes in axial length after posterior scleral reinforcement surgery in 32 eyes

The postoperative changes in the spherical equivalent of refractive error (SE), BCVA, AL, and ERG at the end of follow-up are shown in Table 2. Compared with the preoperative data, the postoperative changes in these variables were statistically significant. The changes in AL outlined in Fig. 1 indicate that although AL was shortened after the surgery, there was an increase in AL during the 6 months after the surgery. However, the mean AL was stable after 6 months, and the final AL reduction recorded was 1.64 ± 0.85 mm.

**Table 2:** The results of preoperative and postoperative examinations

	Preoperative	Postoperative	*P* value
SE (D)	-15.50 ± 8.02	-9.29 ± 7.01	< 0.001
BCVA (logMAR)	0.80 ± 0.49	0.62 ± 0.50	< 0.001
AL (mm)	30.06 ± 1.87	28.42 ± 1.91	< 0.001
IOP (mmHg)	13.37 ± 2.42	13.72 ± 2.08	0.269
ERG a(uV)	33.36 ± 14.10	40.32 ± 17.51	0.005
ERG b(uV)	77.71 ± 28.64	94.05 ± 31.89	0.028

*SE* Spherical equivalent, *BCVA* Best corrected vision acuity, *AL* Axial length, *IOP* Intraocular pressure, *ERG* Electrocardiogram

**Fig. 2:**
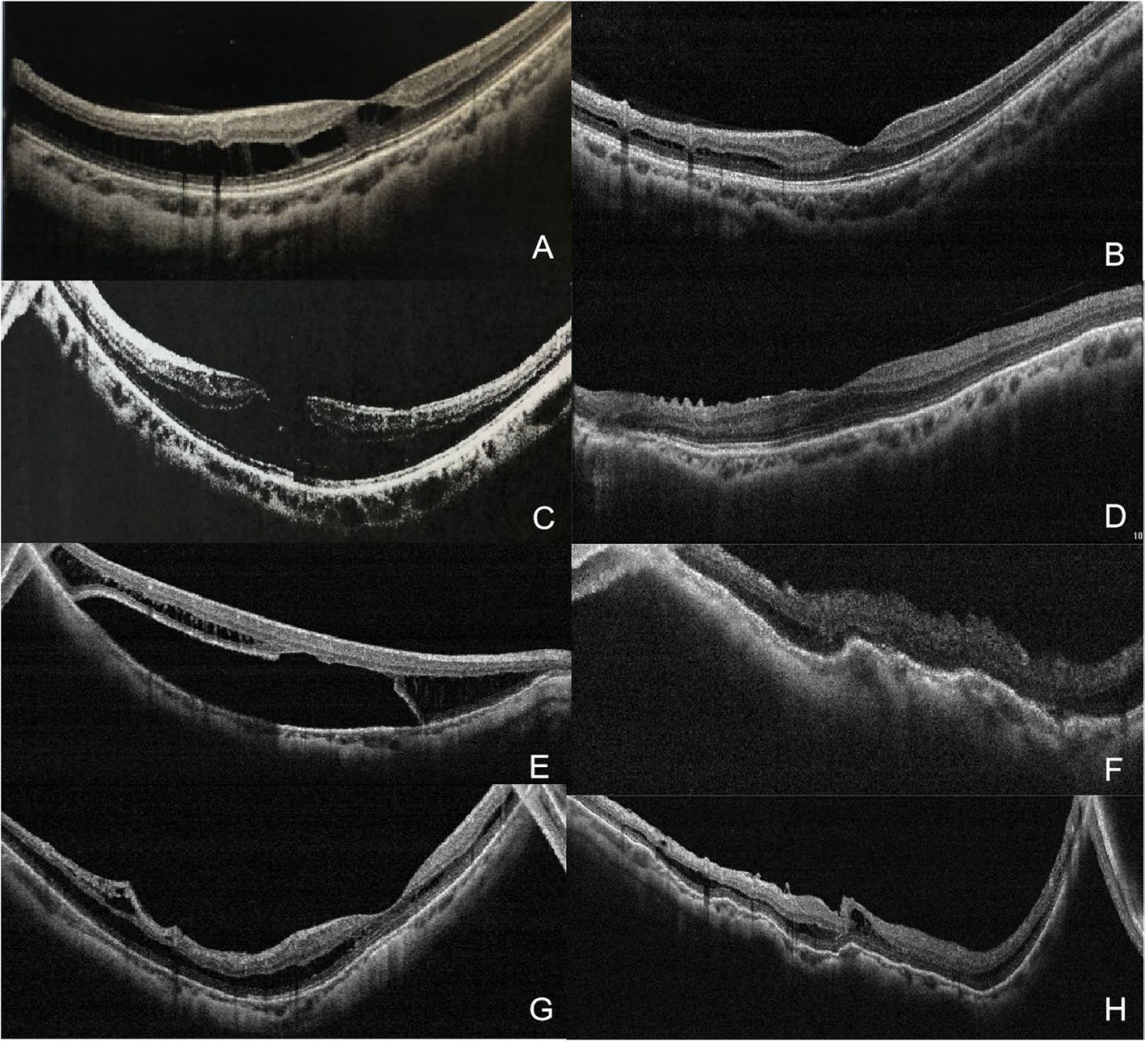
Preoperative and postoperative optical coherence tomography scans of three of the patients who underwent posterior scleral reinforcement. **A** and **B** are the optical coherence tomography scans of Patient No. 3; **C** and **D** are those of Patient No.5; **E** and **F** are those of Patient No.10’s right eye; and **G** and **H** are those of Patient No.10’s left eye

According to the MSS, preoperative OCT scans revealed 1 eye on stage 1, 23 eyes on stage 2, 7 eyes on stage 3, and 1 eye on stage 4, whereas 6 eyes had LMH, 3 eyes had FTMH, as shown in Table 1. Data recorded at the last follow-up showed that the fovea was essentially reattached in 30 eyes (93.75%) and partially reattached in two eyes (6.25%); both PR cases were at stage 3a. The macular holes in seven eyes (77.78%) were closed at the last follow-up, and the two cases in which the MH was not closed were both FTMH. Figure 2 presents the preoperative and postoperative changes in the OCT scans of patients 3, 5, and 10.

Five eyes (15.63%) had transiently high IOP after surgery, which was controlled using IOP-lowering drops, in addition, there was no change in the patient’s visual field after the surgery. All patients had visual distortion but no serious complications such as retinal detachment, choroidal neovascularization and vitreous haemorrhage.

## Discussion

In this study, we assessed the efficacy and safety of PSR for the treatment of MTM. Our results showed a slight increase in AL in the first 6 months after surgery; however, the mean AL stabilised after 6 months. This finding is similar to those reported by Zhu et al. [25]. MTM is the main cause of vision loss in patients with pathological myopia. Although the pathogenesis of MTM is unknown, it is presently considered to be related to two factors, which are as follows [1, 9, 26]: The tangential traction between the posterior vitreous cortex and the inner limiting membrane, and the mismatch between the retina and the externally expanded scleral tissue. The latter is considered to be the main cause of MTM. AL stability is important for the treatment of MTM, as well as for the prevention of recurrence.

Data recorded at the last follow-up in the present study showed that the fovea was in 30 eyes (93.75%) and partially reattached in two eyes (6.25%). Our previous study showed that the essentially reattached rate at 1 year postoperative follow-up was approximately 78.1%-95.8%[5, 27], which were more consistent with this study. We found that both cases of PR were in stage 3a and, as both cases had less than 1 mm of AL shortening at the endpoint of follow-up, we deemed it appropriate to continue to observe the patient if the visual acuity was stable; we reserved a second PSR if the fundus worsened or if the patients experienced vision loss. Several studies [19, 28, 29] have shown reattachment rates of 83.3%–100% for MTM treated using PSR, with variances occurring due to differences in the shape and material of the strips used intraoperatively and due to variations in the duration of the study observations. Micol et al. [7] found that resolution of foveoschisis, retinal reattachment, and MH closure seem to be achieved more frequently with macular buckle than with PPV. The duration of retinal reattachment may be related to the position of the intraoperative strip, the duration and diameter of the foveal retinoschisis, and the shape of the posterior scleral staphyloma. The strip material used in this study was chosen from donor sclerae and sterilised and cross-linked with genipin for increased strength and resistance to degradation, which is beneficial for the prevention of posterior scleral expansion and maintenance of long-term surgical outcomes [30]. The length and width of the strip should be pre-designed before PSR to ensure that it can completely wrap around the macula and posterior scleral staphyloma [5, 11]. The vortex vein, optic nerve, and other important tissues should be avoided while positioning the strip to avoid disrupting the blood supply to the eye and affecting vision. In addition to the genipin cross-linked scleral strips that we applied in PSR, there are several materials and macular buckle shapes, such as L-shape [17] or T-shape [31]. The length and width of our scleral strips can be designed to suit the patient’s AL to ensure that it completely wraps around the posterior scleral staphyloma during surgery. Since we could not observe the patient’s fundus intraoperatively, an OCT could only be performed the day after surgery to ensure that the strips are correctly positioned, and we might adjust the sutures if the results were not satisfactory. However, L-shape [31] did not require posterior sutures or direct access to the posterior pole, and a panoramic system allowed them to obtain the fundus—we believe that this aspect warrants further improvement. The mean duration of follow-up in the study was 17.80 ± 8.74 months, which indicates that the surgical outcomes were stable 1 year after surgery. However, further follow-up is needed to determine the long-term outcomes of the surgery. The MH in seven eyes (77.78%) in the present study closed after surgery. Ikuno et al. [32] reported that the rate of MH closure in patients with high myopia who underwent PPV surgery in their study was 25%. Parolini B et al. [33] suggested that PPV should be performed under appropriate conditions for patients in stage C to reverse the forces exerted by the vitreous in the tangential and perpendicular to the retinal. Both cases are stage c patients; this may be the reason why the MH did not close yet. The two patients in the present study who had no MH closure showed improved visual acuity and retinal reattachment. Considering the visual acuity of the patients and the follow-up time, both patients had improved visual acuity and the follow-up time was 1 year. We believe that we can continue to observe fundus changes. Once visual acuity loss occurs, we can perform PPV surgery for patients in stage C by referring to the treatment principles as Parolini B et al. [33, 34] advised.

Several authors [35, 36] concluded that high myopia causes a decrease in ERG a-wave and b-wave amplitudes in patients and that photopic readings reflect the cellular electrical activities of the retinal layers, from the photoreceptor cells in the macular region to the amacrine cells. Westall et al. [37] concluded that altered retinal cone cell responses appear earlier and are more impaired than those of optic rods in patients with MTM. Therefore, we used photopic ERG to determine whether surgery could offer an improvement in retinal function. Our results showed that the postoperative a-wave and b-wave amplitudes of the patients were significantly improved compared with the preoperative data and that the difference was statistically significant *(p* < 0.05). This indicates that retinal function could be improved using PSR. The preoperative BCVA of the patients in the present study was 0.80 ± 0.49, whereas the postoperative BCVA was 0.62 ± 0.50 (*p* < 0.05). This increase in BCVA may be attributed to the effect of mechanical pressure on the sclera in the posterior pole by the PSR, which can reattach the fovea and delay the increase in AL. Zhang et al. [38] found that both superficial and deep retinal blood flow density and blood flow index in the macular increased after PSR. Zhang et al. [39] found that choroidal thickness and choroidal blood flow increased significantly after PSR in one week. Therefore, we might hypothesize that, due to the stimulating effect of the allograft sclera, neovascularization develops in the sclera, and the blood supply to the corresponding parts of the retina and choroid is improved, promoting metabolism in the optic cells, however, it remains to be proven.

All transient elevations in IOP during the early postoperative period subsided with the use of IOP-lowering drops. The difference between the IOP recorded at the last postoperative follow-up and that recorded during the preoperative period was not statistically significant (*p* > 0.05), suggesting that the shortening of the AL during surgery did not affect the circulation of aqueous humour. There were no changes in the patient’s postoperative visual field indicated that no injury to the vortex vein during the surgery. The postoperative visual distortion is related to the macular fold caused by the PSR, which is a direct confirmation of the effectiveness of the surgery. As the foveal retinoschisis gradually repairs and the folds gradually flatten, the visual distortion progressively improves and eventually disappears. No serious postoperative complications, such as retinal detachment and vitreous haemorrhage, occurred in the present study, indicating the safety of the surgery.

This study has some limitations. First, the observation period was short, and the sample size was small. Parolini et al. [33] performed a follow-up period of 2–8 years and found that different surgical procedures should be given at different stages of MTM. We included patients with MTM after PSR with a short postoperative follow-up, and some patients with poor surgical outcomes may be treated with a combination of PPV when necessary. Second, no control group was established and few indicators of fundus function were evaluated. Considering the complicated pathogenesis of MTM, a large number of long-term clinical studies are needed to verify the long-term efficacy of PSR. In addition, the difference between the efficacy of PSR treatment alone and PSR combined with PPV for MTM needs to be evaluated.

## Conclusion

In conclusion, PSR can achieve anatomical reattachment of the fovea and resolution of retinoschisis by shortening the AL. It can improve retinal function with few postoperative complications, making it a safe and effective method for the treatment of MTM.

## Supplementary Information

**Additional file 1.**

## Data Availability

All data generated or analysed during this study are included in this published article.

## Abbreviations

AL: Axial length
BCVA: Best corrected visual acuity
MTM: Myopic traction maculopathy
MH: Macular holes
OCT: Optical coherence tomography
PPV: Pars plana vitrectomy
PSR: Posterior scleral reinforcement
